# Structural Changes in Primary Teeth of Diabetic Children: Composition and Ultrastructure Analysis

**DOI:** 10.3390/children9030317

**Published:** 2022-02-26

**Authors:** Sadatullah Syed, Syed M. Yassin, Abdulrahman Yahya Almalki, Salma Abubaker Abbas Ali, Abdulaziz M. Maken Alqarni, Yousef M. Moadi, Abdulrahman Masoud Alkhaldi, Nasser M. Alqahtani, Jagadish Hosmani, Artak Heboyan, Shankargouda Patil

**Affiliations:** 1 Department of Diagnostic Sciences and Oral Biology, College of Dentistry, King Khalid University, Abha 61471, Saudi Arabia; sabbas@kku.edu.sa (S.A.A.A.); jhosmani@kku.edu.sa (J.H.); 2 Department of Pediatric Dentistry and Orthodontic Sciences, College of Dentistry, King Khalid University, Abha 61471, Saudi Arabia; syasen@kku.edu.sa (S.M.Y.); aalqarni103@moh.gov.sa (A.M.M.A.); yasiri23@moh.gov.sa (Y.M.M.); abmaalkhaldi@moh.gov.sa (A.M.A.); 3 Department of Preventive Dental Sciences, College of Dentistry, Jazan University, Jazan 25412, Saudi Arabia; ayjalmalki@jazanu.edu.sa; 4 Department of Prosthodontics, College of Dentistry, King Khalid University, Abha 61471, Saudi Arabia; nmalqahtani@kku.edu.sa; 5 Department of Prosthodontics, Faculty of Stomatology, Yerevan State Medical University after Mkhitar Heratsi, Str. Koryun 2, Yerevan 0025, Armenia; heboyan.artak@gmail.com; 6 Department of Maxillofacial Surgery and Diagnostic Sciences, Division of Oral Pathology, College of Dentistry, Jazan University, Jazan 45412, Saudi Arabia

**Keywords:** calcium, phosphorous, diabetes mellitus, dental enamel, type 1, child health, tooth, deciduous, dental caries susceptibility

## Abstract

Diabetes affects the developing enamel by altering the mineralization process, which can have a detrimental effect on oral health. The objectives of this study were to examine the ultrastructure and composition of surface enamel in primary teeth of diabetic children and its clinical implications. Hundred extracted primary teeth from diabetic children (Test group: *n* = 50) and healthy children (Control group: *n* = 50), between 6 and 12 years of age, were subjected to scanning electron microscopy to qualitatively examine the enamel surface. Energy dispersive X-ray (EDX) analysis was performed to investigate the mass percentage of calcium (Ca) and phosphorous (P) in the surface enamel. Ultrastructural aberrations of surface enamel were observed in the test group teeth. Additionally, prism perforations were seen at the junction of rod and inter-rod enamel and the prisms were loosely packed. An even aprismatic layer of surface enamel was evident in the control group teeth. There was a statistically significant difference (*p* < 0.05) of Ca and P mass percentage between the test and control group teeth. The mean mass percentage rates of Ca and P were 33.75% and 16.76%, respectively. A poor surface characteristic and elemental composition of the enamel surface of primary teeth is observed in diabetic children. Therefore, appropriate caries preventive measures are mandatory to maintain the structural integrity of the tooth in these patients.

## 1. Introduction

Type 1 Diabetes (T1D) is a common chronic disorder, caused due to the destruction of insulin-producing pancreatic beta cells. The destruction of beta cells is attributed to autoimmune-mediated pathology affecting the production of insulin necessary for glucose metabolism. Juvenile diabetes (JD) is a type of T1D occurring in children before the age of 15 years, which accounts for five to ten percent of all diabetes cases worldwide [1,2]. Studies have reported moderate to severe consequences of JD in growing children, including those on skeletal maturation, oral and dental health [3,4]. In the oral cavity, diabetes is found to alter the flow rate and buffering capacity of saliva, along with an increase in the salivary glucose level leading to a higher count of Lactobacillus in the planktonic as well as biofilm form [5]. The rate at which caries progresses in diabetic patients is shown to be faster than normal and the defense mechanism of pulp is weaker, culminating in early tooth destruction [6].

In experimental animals, T1D is known to affect the mineralization process and alter the morphological features of enamel, thereby increasing its susceptibility to caries [7]. The detrimental influence of diabetes on amelogenesis leads to deficiencies in the elemental composition, ultrastructure, morphological and mechanical features of mature enamel [8]. A number of morphological changes have been reported in T1D patients, among them, tuberculum interlude and tuberculum sextum are the most common [9]. Several studies utilizing macroscopic and microscopic observations have so far been able to demonstrate the effects of diabetes on developing dental hard tissues [10,11,12]. The frequently encountered enamel defects include modified ultrastructure, whitish fields, degenerative lesions and hypoplastic enamel [13,14,15]. Moreover, diabetes-induced disequilibrium between demineralization and remineralization of enamel in the presence of altered ecology of the oral cavity results in a significant increase in caries risk [15,16].

Energy Dispersive X-ray (EDX) analysis of enamel of diabetes-induced rats has shown a reduction in the concentration of calcium (Ca) and phosphorous (P) [7,17]. Low levels of Ca in presence of hyperglycemia are attributed to a decrease in the ameloblast activity during amelogenesis [11]. An alternate hypothesis for the compositional and structural changes in diabetic enamel is due to the disturbance in ion exchange in cellular metabolism associated with diabetes [18]. 

The effect of diabetes on enamel ultrastructure, to date, has been tested only on rodents. None of the previous studies have examined the ultrastructure and composition of enamel in human primary teeth of diabetic children. Therefore, the objectives of this study were to analyze the structural properties of surface enamel in primary teeth of diabetic children. The study also aimed to assess any changes in the elemental composition in the same teeth. The clinical significance of the ultrastructural and compositional changes and the preventive measures to reduce caries risk in these patients were also explored. 

## 2. Materials and Methods

### 2.1. Sample Collection 

This prospective study was carried out over a period of 19 months between 2018 and 2020 in the Aseer Dental Center and Pediatric Dental Clinics of King Khalid University College of Dentistry. Before commencing the study, ethical approval was obtained from the King Khalid University College of Dentistry Institutional Review Board with approval letter number SRC/ETH/2016-17/049. Informed consent from the parents was obtained for each child included in the study.

The study subjects were screened on the basis of the American Academy of Pediatric Dentistry Caries Risk Assessment Tool to make sure the biological, social, protective and clinical factors were similar for both the test and control groups [19]. In total, 50 extracted primary teeth from children diagnosed with diabetes aged 6–12 years were included in the test group. The extraction protocol of the test group was based on the guidelines for the management of children and adolescents with diabetes requiring surgery [20]. Since the study required normal crown anatomy, we excluded carious, restored, structurally defective teeth, and teeth from children suffering from other systemic diseases.

Simultaneously, 50 primary teeth of healthy children matching the age of the test group and fulfilling the inclusion criteria were used as the control group. The reason for tooth extraction in the study samples was either pre-shedding mobility or retaining the primary tooth.

### 2.2. Sample Preparation and SEM–EDX Analysis 

After extraction, the teeth were disinfected with formalin, coded, and stored in normal saline at room temperature during the collection period. Prior to SEM imaging, the teeth were washed with fresh normal saline solution and pat-dried with sterile cotton gauze. 

The crown surface was subjected to SEM analysis using a JEOL JSM-636OLA Analytic Scanning Electron Microscope (JEOL, Tokyo, Japan) with backscatter electron mode. The SEM was operated at a voltage between 15 and 25kV. The scanning site was standardized for all of the teeth samples by focusing on the mid-labial/buccal aspect of the crown. The enamel was visualized under the microscope, and the images were saved in jpg file format. Ultrastructural characterization of enamel prisms and crystal deposition pattern of the enamel surface was determined by detailed visual examination of the images in Windows Microsoft Office Picture Manager Software (Microsoft Inc., 2016, Redmond, WA, USA). 

Following SEM imaging, enamel surface composition analysis was performed in the same areas of the crown using EDX Spectrometry. The same SEM unit (JEOL JSM-636OLA Analytic Scanning Electron Microscope) was used to record the mass percentage rates of Ca and P. The mean mass percentage rates of Ca and P in the test and control groups were statistically analyzed by independent *t*-test using STATA Version 9.2. The statistical significance was set at 5%. The clinical significance of the study findings was interpreted based on the outcomes of previous reports on altered enamel in primary teeth.

## 3. Results

### 3.1. SEM Analysis 

**Figure 1:**
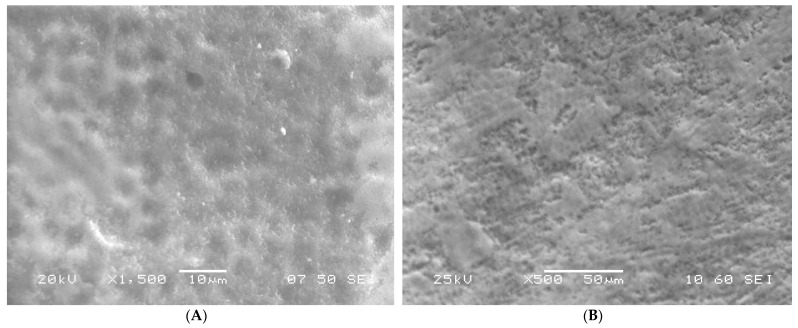

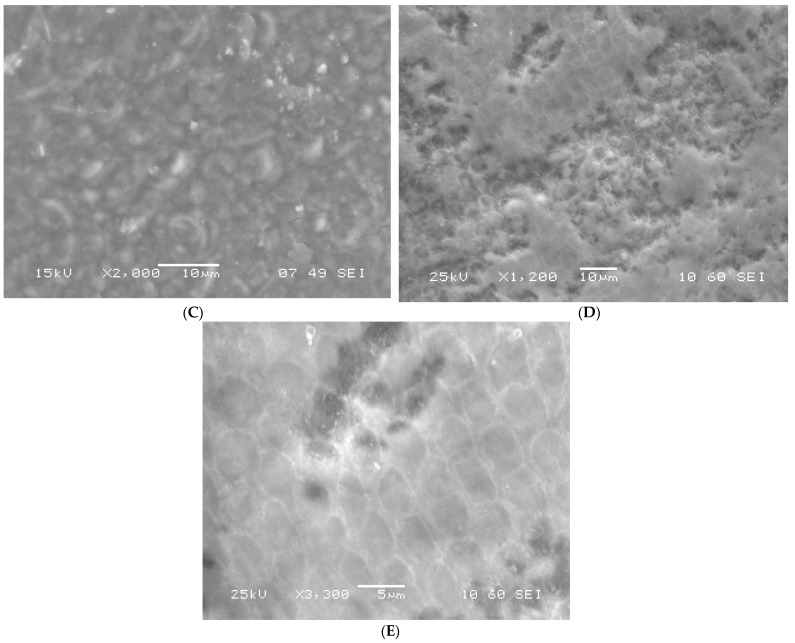
(**A**) SEM image of the labial surface of primary tooth showing irregular enamel in a diabetic patient; (**B**) SEM image of the labial surface of primary tooth showing enamel surface with normal ultrastructure in a healthy child; (**C**) SEM image of the labial surface of primary tooth showing aprismatic enamel in a diabetic patient; (**D**) SEM image of the labial surface of primary tooth showing enamel surface with a normal aprismatic layer in a healthy child; (**E**) SEM image of the labial surface of primary tooth showing enamel surface with a normal rod and inter-rod structure visible beneath the aprismatic layer in a healthy child.

**Figure 2:**
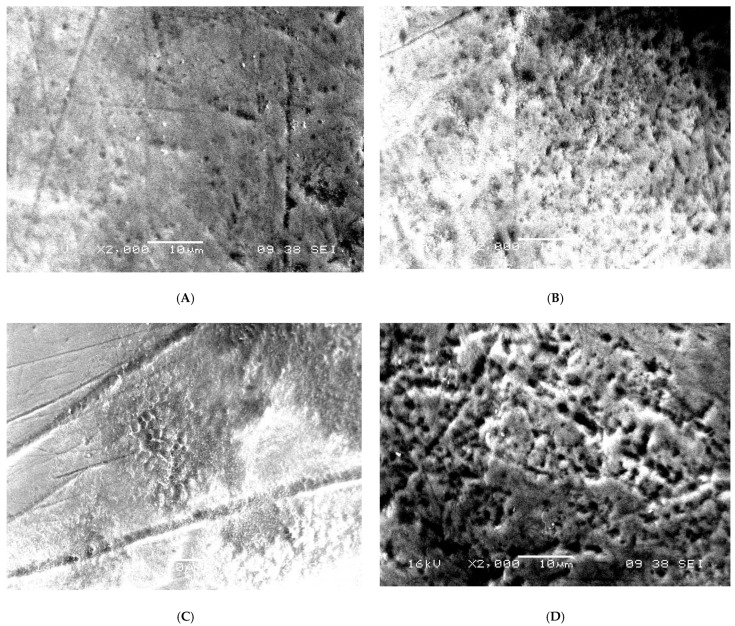
(**A**) SEM image of the labial surface of primary tooth showing normal enamel surface with perforations and craze line-like depressions in a diabetic patient; (**B**) SEM image of the labial surface of primary tooth showing enamel surface with perforations in a diabetic patient; (**C**) SEM image of the labial surface of primary tooth showing enamel surface with perforations and deep craze line-like depressions in a diabetic patient; (**D**) SEM image of the labial surface of primary tooth showing enamel surface with numerous perforations.

**Figure 3:**
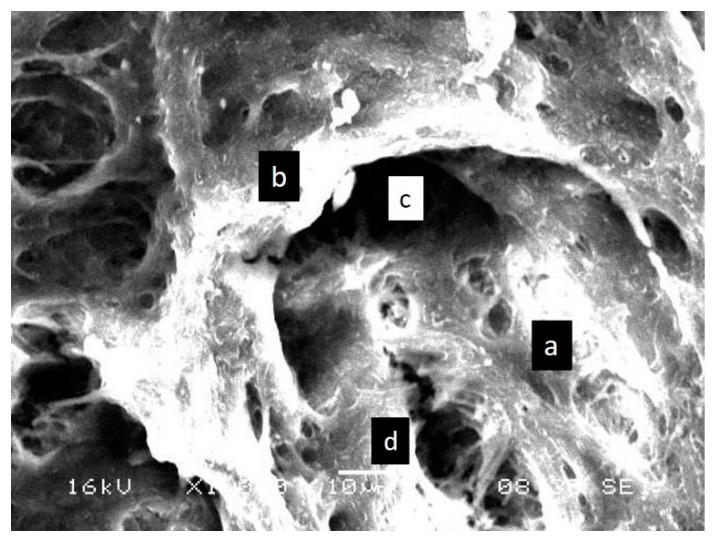
SEM image of the labial surface of primary tooth showing loosely packed enamel rod and inter-rod: (**a**) enamel rod; (**b**) inter-rod enamel; (**c**) rod sheath space; (**d**) perforations along the rod.

Ultrastructural aberrations were observed in the SEM images of the enamel surface of teeth in the test group. An uneven aprismatic layer (Figure 1A,C) with numerous perforations and deep craze line-like depressions (Figure 2A–D) along the surface were the common aberrations observed. The enamel surface in the test group was composed of a false-type aprismatic enamel. At higher magnification, loosely packed enamel rods with perforations at the junction of rod and inter-rod enamel were evident (Figure 3). Compared with the abnormalities observed in the test group teeth, the enamel surface of control group teeth demonstrated an even aprismatic layer. With higher magnification, normal rod and inter-rod enamel structures were visible below the aprismatic layer (Figure 1B,D,E). 

### 3.2. EDX Analysis

**Figure 4:**
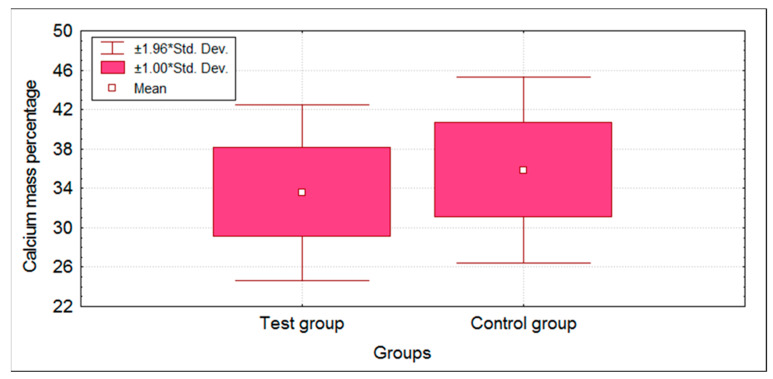
Box plot showing mean Calcium mass percentage scores of test and control groups. * standard deviation.

**Figure 5:**
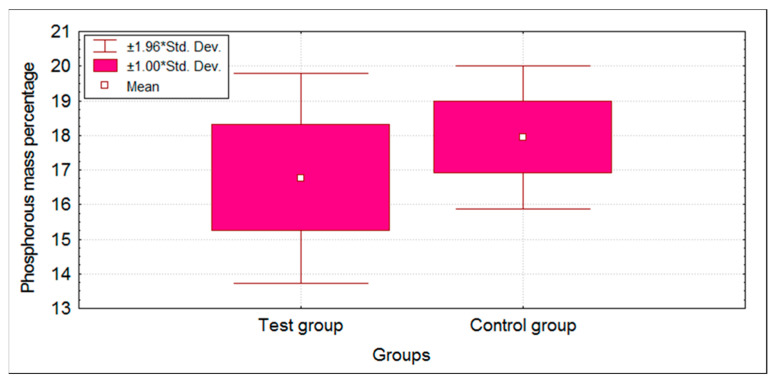
Box plot showing mean Phosphorous mass percentage scores of test and control groups. * standard deviation.

EDX analysis demonstrated changes in the mass percentage of Ca and P in the test group compared to the control group. The mass percentage values in both groups followed a normal distribution; therefore, a parametric independent *t*-test was applied. There was a statistically significant difference (*p* < 0.05) in the mass percentage of Ca and P between test and control groups (Table 1). A decrease in the mean mass percentage of Ca and P was observed in the test group when compared with the control group (Figure 4 and Figure 5). 

## 4. Discussion

In the present study, changes in ultrastructure and composition of enamel were observed using backscattered SEM and EDX spectroscopy. SEM is a powerful magnification tool with several advantages for analyzing the ultrastructure and elemental composition of dental hard tissues. It provides quick and detailed morphological and compositional data with minimal sample preparation. It allows the examination of surface morphology at the nanometer level leading to high-resolution digital images. The EDX spectroscopy attachment along with the backscattered SEM serves as a useful combination in examining elemental composition at the micron level.

The SEM results of this study confirm the presence of ultrastructural changes in the surface enamel of primary teeth of diabetic children. The most common defect observed in the test group SEM images were loosely packed prisms on the surface of the enamel. Enamel is loosely packed primarily due to the lack of enough mineral content and alteration in the morphology and structural pattern of hydroxyapatite (HAP) crystals. Similar changes are reported in the experimental research on Wistar rats with artificially induced diabetes, where the changes in enamel and its mechanical features are comparable [15,17]. Metabolic disturbances during the pre-natal and post-natal periods are known to leave an impression on the developing hard tissues of teeth. The neonatal line is a good example of such an effect on the structure of both enamel and dentin [21]. JD manifests in children approximately around the same time when amelogenesis of primary teeth (14–18th week of gestation) and permanent teeth (28–32nd week of gestation) begins [22].

While amelogenesis taking place under the influence of JD is a significant event, several other factors collectively may lead to defects in the enamel. For example, the lack of proper proteinases activity can affect amelogenesis and lead to loosely packed prisms in mature enamel. The development of enamel begins with high protein content and ends in high mineral content [23], with most of the protein being replaced by minerals through proteinases activity [24]. The proper establishment of HAP crystals in the rod and inter-rod enamel is an intricate and complex process dependent on the precise expression of the relevant proteinases. Brings et al. [25] reported a change in matrix metalloproteinase (MMPs) in experimental diabetic rats associated with altered collagen metabolism. MMPs are proteinases, capable of degrading all kinds of extracellular matrix proteins [26]. Matrix metalloproteinases-20 (MMP-20) are the proteinases expressed during the secretory stage and early maturation stage of amelogenesis, wherein they cleave the enamel matrix proteins resulting in a mold which establishes the size and shape of the developing HAP crystal. Once the HAP crystals grow to their full size and amelogenesis advances to the maturation stage, another proteinase, kallikrein-related peptidase-4 (KLK4), cleaves the mold allowing the maturing enamel to interlock and form a highly mineralized interconnected crystal structure [24]. Perturbations in this genetically programmed sequence of events, caused by hyperglycemia and oxidative stress, may theoretically result in altered ultrastructure of mature enamel as seen in SEM images of our test samples.

The other common ultrastructural defect observed in the SEM images of diabetic primary teeth was an uneven aprismatic layer with surface perforations. The normal surface of enamel exhibits an even, highly mineralized aprismatic or prismless layer formed by the shortened ameloblast in the late secretory stage of amelogenesis, as evident from the control samples. Kodaka et al. [27] categorized aprismatic enamel into false type, moderate type, essential type, and complex type aprismatic enamel. While all the types, except the false type, are considered aprismatic enamel, the type observed in the test samples was the false type. This is so because the surface was either completely devoid of aprismatic enamel or sparse islands of aprismatic enamel were seen. The numerous surface perforations we observed in the test group images are likely due to the disturbed ameloblast activity leaving indentations negative to the shape of the tomes process of the ameloblast cell during a secretory stage. 

In our study, the mass percentage of Ca and P in the test group teeth were significantly lower than the control group teeth. According to experimental data, the disturbance in homeostasis in diabetes causes dyselectrolytemia and transmineralization leading to changes in the mineral components of bone and teeth [28]. During amelogenesis, this is manifested as disruption of the transport of ions such as Ca and P between ameloblasts and its secreted extracellular matrix. Ca and P are the two main inorganic components of HAP crystals, which together with other similar minerals make up 96% (by weight) of mature enamel [29]. The EDX findings of our study validate the above concept. Moreover, the average concentration of Ca and P in the control group teeth is comparable to the findings of Armstrong W and Brekhus PJ [30]. 

In short, we report defects in the enamel surface of primary teeth in diabetic children, akin to the defects reported by several studies on diabetes-induced experimental rats. Abnormal ameloblast activity appears to be the prime reason for the changes observed in the enamel surface. The following two hypotheses may be indicative of the changes brought about by the ameloblasts.

1. The effect of hyperglycemia on the mineralization leads to a low concentration of Ca and P in the mature enamel. Decreased levels of Ca and P could be a direct reason for the enamel defects. The work of Atar et al. [31] supports this hypothesis of ours.
2. In addition to the impact on elemental composition, the influence of oxidative stress on the expression of MMP-20 and KLK4 during the secretory stage can lead to impaired development of HAP crystals in the rod and inter-rod enamel. The exact effect of diabetes on MMP-20 and KLK4 is still unreported, however, our assumption in this regard is based on the literature published by Bartlett JD [24].

Dental Caries is the most significant threat that can be caused due to defects observed in the surface enamel of diabetic children. Defective surface increases the risk of acid dissolution of surface enamel, leading to caries. Special caries prevention protocol seems mandatory to maintain caries-free teeth in the already compromised oral condition of diabetic children. By special prevention protocol, we mean steps that are specific to high caries risk children, such as the use of topical remineralizing agents including fluorides, and active measures by parents and oral health professionals aimed at maintaining the non-cariogenic oral environment and monitoring possible caries development.

Considering the results of this study alongside the data available regarding the enamel of T1D teeth we recommend the following measures to reduce the risk of caries in diabetic children.

- Primary teeth of diabetic children should be screened for macroscopic deficiencies as soon as they erupt.
- Diabetic children should be considered high caries risk children and they should be clinically monitored every three months.
- Primary and secondary prevention protocols specific to the teeth of diabetic children should be developed and implemented to curtail the initiation of dental caries.
- The parents of diabetic children should be made aware of the increased risk of dental caries and its consequences at an early stage.
- The parents should be educated to ensure these children maintain good oral hygiene and a healthy non-cariogenic diet.
- Finally, parents should be counseled to help maintain healthy blood glycemic levels to prevent the undesired oral and systemic consequences of diabetes.

## 5. Conclusions

Poor ultrastructural surface characteristics of enamel surface are observed in primary teeth of diabetic children. Abnormal amelogenesis activity in primary teeth of diabetic children seems to be the cause of these defects in the enamel surface. The defects make the enamel more prone to acid dissolution, thus increasing caries risk. Therefore, appropriate caries preventive measures are mandatory to maintain the structural integrity of enamel in these patients.

## Figures and Tables

**Table 1:** Comparison of mean mass percentage of Ca and P between test and control groups.

	Groups	*n*	Mean	SD	SE	t-Value	*p*-Value
Ca	Test group	50	33.57	4.56	0.65	−2.4232	0.0172 *
	Control group	50	35.84	4.83	0.68		
P	Test group	50	16.76	1.55	0.22	−4.4569	0.0001 *
	Control group	50	17.94	1.05	0.15		

* *p* < 0.05.

## Data Availability

Not applicable.

## Sample Availability

Samples of the teeth are available from the authors.
